# Pre-Analytical Factors that Affect Metabolite Stability in Human Urine, Plasma, and Serum: A Review

**DOI:** 10.3390/metabo9080156

**Published:** 2019-07-25

**Authors:** Victoria L. Stevens, Elise Hoover, Ying Wang, Krista A. Zanetti

**Affiliations:** 1 Behavioral and Epidemiology Research Group, American Cancer Society, Atlanta, GA 30303, USA; 2 Epidemiology and Genomics Research Program, Division of Cancer Control and Population Sciences, National Cancer Institute, Rockville, MD 20850, USA; 3 PKD Foundation, Kansas City, MO 64131, USA

**Keywords:** metabolomics, metabolite, pre-analytical factors

## Abstract

Metabolomics provides a comprehensive assessment of numerous small molecules in biological samples. As it integrates the effects of exogenous exposures, endogenous metabolism, and genetic variation, metabolomics is well-suited for studies examining metabolic profiles associated with a variety of chronic diseases. In this review, we summarize the studies that have characterized the effects of various pre-analytical factors on both targeted and untargeted metabolomic studies involving human plasma, serum, and urine and were published through 14 January 2019. A standardized protocol was used for extracting data from full-text articles identified by searching PubMed and EMBASE. For plasma and serum samples, metabolomic profiles were affected by fasting status, hemolysis, collection time, processing delays, particularly at room temperature, and repeated freeze/thaw cycles. For urine samples, collection time and fasting, centrifugation conditions, filtration and the use of additives, normalization procedures and multiple freeze/thaw cycles were found to alter metabolomic findings. Consideration of the effects of pre-analytical factors is a particularly important issue for epidemiological studies where samples are often collected in nonclinical settings and various locations and are subjected to time and temperature delays prior being to processed and frozen for storage.

## 1. Introduction

Metabolomics provides a comprehensive assessment of numerous small molecules in biological samples. As it integrates the effects of exogenous exposures, endogenous metabolism, and genetic variation, metabolomics is particularly attractive for studies seeking to find metabolic profiles associated with a variety of chronic diseases including cardiovascular diseases, diabetes, and cancer. Increasingly, epidemiological studies are using metabolomics to search for metabolites that reflect specific exposures [1], are an early marker of disease [2], represent pathways that contribute to disease development [3], or are useful for predicting risk of disease [4]. 

Metabolomic analyses are sensitive to variability in sample handling and preparation. This is a particularly important issue for epidemiological studies where samples are often collected in nonclinical settings and different locations and are subjected to delays of various times and temperatures before being processed and frozen for storage. Specific knowledge of how differences in the type of sample, sample collection conditions, sample processing, and sample storage influence metabolite levels is needed for metabolomic studies to maximize sensitivity and specificity and provide robust and reproducible results. For studies where samples have not yet been collected, knowledge of the effects of pre-analytical factors on metabolomic assessments can be used to optimize the collection process while, in studies where samples have already been collected, this knowledge may indicate which metabolites among those analyzed are likely to be affected by collection conditions and should be considered for exclusion from the study. 

In this review, we examined studies that have characterized the effects of various pre-analytical factors on both targeted and untargeted metabolomic studies. Previous reviews have investigated some of these factors as they relate to studies using blood fractions [5,6] or untargeted metabolomic approaches [7] and many are described in a recent publication describing best practices for biological samples for metabolomics research [8]. Here, we focus on studies of serum, plasma, and urine, which are the most common sample types used in epidemiological studies. 

## 2. Results 

**Figure 1:**
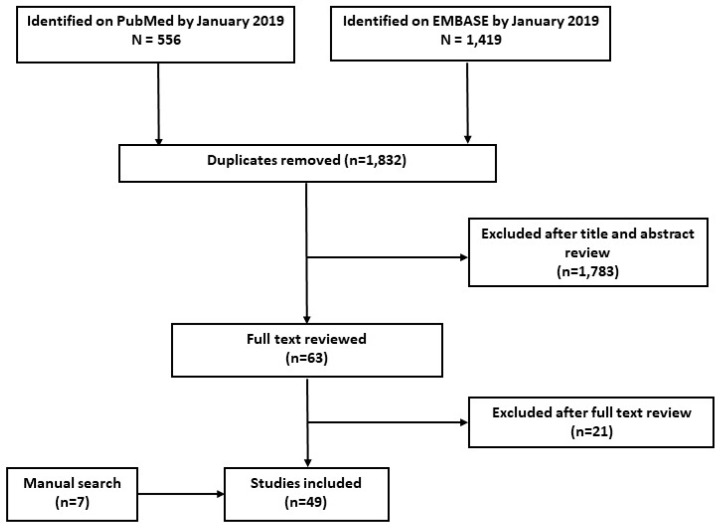
Inclusion schema.

Figure 1 shows the article inclusion schema. A total of 556 articles were identified on PubMed and 1419 on EMBASE as of 14 January 2019. Of those articles identified, 1832 were determined to be duplicates and therefore removed from the review. Of the remaining articles, 1783 were excluded after title and abstract review, resulting in 63 articles. Additionally, seven articles were identified by manual search and included. 

In total, 66 studies were reviewed in detail and 49 were found to meet the inclusion criteria (see Materials and Methods). Of these, 35 examined pre-analytical factors in blood samples only (71.4%), 10 used urine samples exclusively (20.4%), and four used both blood and urine samples (8.2%). The majority of the studies used a mass-spectrometry (MS)-based analytic platform (77.6%) and untargeted metabolomic methods (69.4%).

### 2.1. Blood Samples

**Figure 2:**
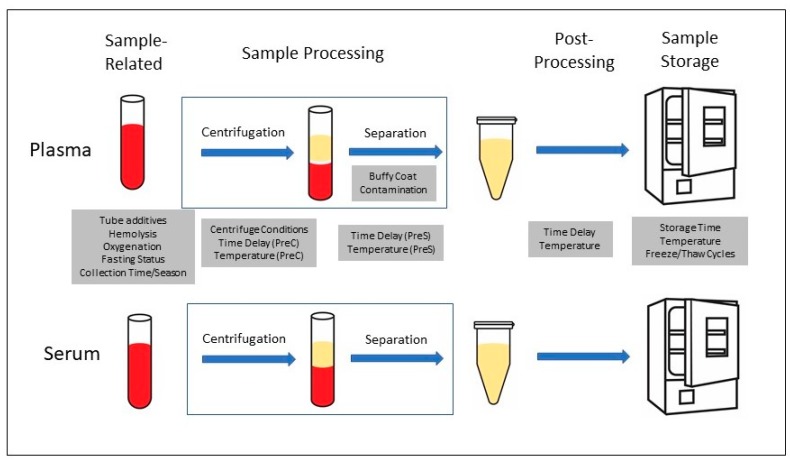
Pre-analytical variables for plasma and serum samples.

The most common blood fractions used in metabolomic studies are plasma and serum. These are prepared from whole blood using standard processes illustrated in Figure 2. The pre-analytical variables that have been investigated are shown in the shaded boxes either below (for plasma) or above (for serum) the step to which they relate. In addition, whether plasma or serum is better for metabolomic studies has been addressed. Individual metabolites found to be affected by various conditions in specific studies are listed in Table S1 in Supplementary Materials.

Table 1 summarizes the studies of pre-analytical factors that influence metabolomic studies conducted with blood biospecimens and the results are described below.

Serum Versus Plasma: Whether serum or plasma is better for blood metabolomic studies has been addressed by direct comparison of metabolomic profiles from the two blood fractions as well as by the sensitivity of each to various pre-analytical variables. Seven studies directly investigated differences in metabolomic findings from serum and plasma samples [9,10,11,12,13,14,15]. Comparing nuclear magnetic resonance (NMR)-derived metabolomic profiles from four independent samples, Teahan et al. [9] found that differences between serum and plasma were minimal. Wedge et al. [12] also found small differences between the MS-based metabolomic profiles from the two blood fractions, but judged plasma to be superior because correlations between specific metabolites and life expectancy among 29 small-cell lung cancer patients were found using plasma but not using serum samples. Denery et al. [15] found that serum samples from 12 donors analyzed using MS-bases platforms had higher levels of some peptides and protein fragments than plasma, but levels of lysophosphatidylinositol were higher in plasma than in serum. The other four studies, which used MS-based platforms, found that the levels of many of the metabolites measured were higher in serum than in plasma [10,11,13,14]. Both Nishiumi et al. [10], who used an untargeted approach, and Paglia et al. [11], who used a targeted approach, suggested that serum may provide higher sensitivity than plasma. Yu et al. [13] and Breier et al. [14] also found higher levels of metabolites in serum but focused on reliability rather than sensitivity. Based on finding a mean correlation coefficient for 163 metabolites of 0.83 for plasma and 0.80 for serum, Yu at al. [13] concluded that plasma provided more reproducible metabolomic profiles. Breier et al. [14] found that the median interclass correlation coefficient for 159 metabolites was 0.66 in serum and 0.63 in plasma and suggested that serum results had higher reliability. Thus, these studies suggest that both serum and plasma are appropriate for metabolomic research but may differ in terms of sensitivity and reliability.

Three studies have investigated whether metabolomic profiles from serum and plasma samples differed in their response to delays in sample processing (pre-centrifugation) [16,17,18], post-processing [17,19] and freeze/thaw cycles [17]. Bernini et al. [19] tested the effects of a 4-h processing delay at either 4 °C or room temperature and a 2-h post-processing delay at room temperature and found that the NMR-derived metabolomic profiles from plasma was slightly more stable to both delays than those from serum. Kamlage et al. [18] investigated the effect of room temperature processing delays of varying lengths on the levels of two metabolites, taurine and O-phosphoethanolamine, that were measured using an untargeted MS-based metabolomic approach. Levels of both metabolites were significantly affected after a shorter time delay in serum than in plasma, consistent with having higher stability in plasma. Finally, Hirayama et al. [17] compared the effects of processing delays of up to 3 h, post-processing delays up to 3 h and up to 10 freeze/thaw cycles on serum and plasma samples from four volunteers. NMR-derived metabolomic profiles from plasma were found to be more stable to the processing delay than were those from serum. However, this delay was at 4 °C for the plasma samples and at room temperature for the serum samples, which may account for the differences observed. The post-processing delay, which was at either 4 °C or room temperature for both sample types did not reveal any differences. However, more metabolites were affected by five or 10 freeze/thaw cycles in the serum samples than in the plasma samples.

### 2.2. Blood Sample Collection

Tube Additives: The effects of tube additives as they apply to both plasma and serum samples have been tested. For plasma, three studies tested the effect of heparin versus ethylenediaminetetraacetic acid (EDTA) on the overall metabolomic profile as measured by NMR [20] or metabolite variability [21] or stability [22] using MS-based platforms. Pinto et al. [20] found that the metabolomic profile was unaffected by whether the tube additive was heparin or EDTA. Double the normal heparin concentration also did not significantly affect metabolic profiles measured by NMR [23]. Townsend et al. [21] reported that 92% of the 158 metabolites measured had similar variability regardless of tube additive and Midttun et al. [22] found that the stability of most of the 38 metabolites measured was similar in EDTA- and heparin-plasma. In addition to comparing plasma from heparin- and EDTA-containing tubes, Hebels et al. [24] also tested citrate-containing tubes and Yin et al. [25] tested both citrate- and sodium fluoride-containing tubes. Hebels et al. [24] reported none of the additives introduced any additional variation in the MS-derived metabolomic profiles, but concluded that studies should not compare plasma prepared with different tube additives. Yin et al. [25] found that plasma from heparin- and citrate-containing tubes had more noise in the spectra than did that from EDTA- or sodium fluoride-containing tubes. Denery et al. [15] found only small differences in the metabolomic features measured using MS-based platforms in plasma from 12 donors prepared from either heparin- or citrate-containing tubes. Paglia et al. [11] compared plasma from EDTA and citrate containing tubes and reported small differences in MS-derived metabolomic profiles. Finally, Malm et al. [26] compared EDTA-containing tubes with and without a gel separation plug and found no effect on the MS-derived metabolomic profiles of the plasma samples. Thus, these studies provide no strong evidence that any tube additive for plasma preparation is superior to the others for either NMR- or MS-based metabolomic analyses.

For serum, only a single study has tested the effect of tube additives on metabolomic analyses. Breier et al. [14] compared serum from tubes with and without a gel barrier and found that only one of 188 metabolites measured in a targeted MS-based analysis differed between samples. Thus, whether the collection tubes contain a gel barrier does not affect metabolomic findings on serum samples.

Fasting Status: The influence of fasting status on blood metabolomic measures has been evaluated in seven studies. Two untargeted studies of 16 participants using NMR [20] and 26 participants with MS [39] found that fasting contributed little to the variability of the overall metabolomic profile compared to other factors such as sample preparation. In a targeted MS-based study of 66 subjects in which 158 metabolites were measured, Carayol et al. [32] found that fasting status affected amino acids, hexoses, and some lipids minimally, but altered the levels of some other lipids more significantly. In an untargeted MS study of similar size and metabolite number, Townsend et al. [21] found that the median difference in peak areas between fasting (>8 h since eating) and non-fasting samples was 1–6% for various metabolite classes. Two larger untargeted MS studies [42,44] reported that fasting status significantly affected specific classes of metabolites more than others [44] and 34% of the metabolites measured [42]. Finally, a targeted MS-based study of serum from 40 subjects showed that fasting status affected levels of several amino acids and acylcarnitines [43]. Overall, these studies indicate that many metabolites are not affected by fasting status and, those that are sensitive to this can be identified and, if needed, be excluded from further analyses.

Hemolysis: The impact of hemolysis on blood metabolomic measures has been evaluated in three studies. Each assessed hemolysis using Grade 1 (moderate) and Grade 2 (severe) hemolysis. Two studies [25,38] found that hemolysis had significant effects on the MS-derived plasma metabolome. With samples from 20 participants, Kamlage et al. [38] found that the number of metabolites affected increased with hemolysis level, with 18% changed in Grade 1 and 30% changes in Grade 2 hemolysis. Yin et al. [25] showed a heat map of 69 metabolic features that were significantly changed by hemolysis. In contrast, hemolysis had little effect on metabolomic profile derived using NMR with plasma in 20 patients [23]. Thus, hemolysis appears to adversely affect metabolomic study done with MS but not NMR. This difference may be related to lower sensitivity of NMR analyses relative to that of MS-based platforms [23].

Oxygenation: To determine if differential exposure to oxygenation influenced the metabolomic profile, Bervoets et al. [23] exposed samples to oxygen during tube transfer. The MS-derived metabolomic profile was unaffected by excess oxygen exposure. 

Collection Time of Day and Season: Three untargeted MS studies examined the effect of collection time during day on metabolomic findings. Townsend et al. [44] found little variation in metabolites levels by time of day of plasma sample collection but recommended consistent collection times for studies looking at bile acids and vitamin metabolites levels, two metabolite categories found to be sensitive to this variable. Kim et al. [39] found that time of day had little effect on the plasma metabolomic profile. Finally, Ang et al. [28] found that 203 (19%) of 1069 metabolite features varied in plasma samples for eight men collected at different times of the day. The metabolites altered included corticosteroids, bilirubin, amino acids, acylcarnitines, and phospholipids. 

Townsend et al. [44] also examined differences in metabolite levels by season of blood collection. Most metabolites were unaffected but some, including bile acids, organic acids, purines and pyrimidines, differed in months with peak sun (May–October) as compared to low sun (February–April). These findings suggest that varying time of day or season of blood draw only affect the metabolomic analyses of specific metabolites. 

### 2.3. Blood Processing

Time Delay and Temperature (Pre-Centrifugation): Numerous studies have investigated the influence of delays of various times and temperature between blood collection and centrifugation to yield either serum or plasma on metabolomic profiles. In addition, while time and temperature may seem to be separate variables, the influence of the time delay varies depending on the temperature. Therefore, these two variables are discussed together.

The 20 studies that investigated pre-centrifugation processing delays included six studies that used untargeted NMR analyses [9,19,23,31,34,37], four studies that used targeted MS-based platforms [14,17,22,47], nine that used untargeted MS-based platforms [10,18,21,24,25,33,36,45,46], and one study that used both targeted and untargeted MS-based platforms [38]. The number of time points tested varied from one [33] to five [25] and the durations tested ranged from less than an hour [9,10,23] up to 48 h [22,46]. The temperatures evaluated fell into two groups. The first, which ranged from 0 to 4 °C, was achieved using ice or cold packs [14,21,22], wet ice [9,19,23], or refrigeration [10,17,25,31,33,34,36,37,38,46,47]. The second temperature group was intended to represent room temperature and ranged from 18 to 25 °C [9,10,18,24,25,31,33,34,37,38,45].

Overall, metabolite levels and metabolomic profiles were shown to change more rapidly when the processing delay was at room temperature than at cold temperature [9,10,19,25,31,34,38,45]. This was observed with blood that was subsequently processed to both serum and plasma and was independent of the metabolomic platform. The shortest processing delay at room temperature, that was examined, was 15 min. With samples from three volunteers, Nishiumi et al. [10] found a few metabolites were altered after just a 15 min processing delay of plasma samples and many more were changed after 30 min. All the other studies, which included up to 96 samples [37], also reported significant effects of pre-centrifugation processing delays at room temperature [9,14,17,18,19,25,26,31,34,37,38,45]. These findings indicate that processing delays at room temperature should be avoided.

The findings regarding processing delays at 0–4 °C vary somewhat depending on how the metabolomic data was evaluated. Dunn et al. [33] concluded that the variance caused by a 24-h processing delay at 4 °C was similar to that of serum processed with no delay whereas others report significant effects on metabolomic profiles with shorter delays at cold temperature [24,25,31,34,37,45]. The main difference that appears to account for the difference in findings is that Dunn et al. [33] used multivariate analyses to characterize the variance in 200 peaks measured in an untargeted MS-based analysis while most of the other studies included some comparisons of individual metabolites levels whose chemical identity was known. 

In addition to characterizing the effect of pre-centrifugation processing delays and temperature on metabolite profiles and levels, several of the studies compared the variance introduced by these pre-analytical variables to the overall variance found between samples from different individuals. Regardless of metabolic platform or approach or whether serum or plasma, the conclusion from all these studies was that the between-person variability was greater than that due to processing time delays and temperature [9,17,22,46].

Many of the studies of processing time and temperature effects investigated which metabolites were most sensitive to these parameters [9,10,14,17,18,21,22,23,25,31,34,36,37,38]. These varied depending on the specifics of metabolomics platform, but several studies found energy-related metabolites such as glucose, lactose, and pyruvate to be among the most labile metabolites [9,23,34,36,37,38,46]. Three of these studies have used their findings to develop tests to determine whether samples may have been exposed to problematic processing conditions [23,36,45] and Brunius et al. [31] have developed a model to minimize processing condition-derived variance in NMR-derived metabolomic data.

Centrifugation Conditions: The impact of centrifugation conditions on the metabolite profile has been evaluated in four studies. Investigators looked specifically at centrifugation speed, temperature, time, and brake force. With a single pooled sample, Ammerlaan et al. [27] examined all four aspects in both serum and plasma and found that variation in speed, time, and brake force did not significantly affect metabolomic profiles. Serum and plasma samples from blood centrifuged at 4 °C versus 20 °C varied in some characteristics, but there was minimal difference in the levels of specific metabolites in the samples. Similarly, Jobard et al. [37] investigated all but brake force on both serum and plasma samples from 96 individuals and reported that variation in centrifugation speed, temperature, and time did not alter the metabolomic profiles obtained with NMR from these samples [40]. 

The other two studies investigated fewer centrifugation conditions and only used plasma. Bervoets et al. [23] only varied temperature saw no impact between centrifugation at 4 °C versus room temperature on metabolomic profiles in plasma from 20 people. Lesche et al. [40] compared centrifugation at 1500× *g* for 10 min to 3000× *g* and found significant differences in the NMR-derived metabolomic profiles of resulting plasma samples from 10 donors. The authors did not conclude that either of these conditions were superior, but recommended standardization of centrifugation conditions for metabolomic studies. 

Time Delay and Temperature (Post-Centrifugation): How a delay in removing either the serum or plasma fraction may influence metabolomic results was investigated in one study. Malm et al. [26] compared metabolomic profiles and metabolite levels measured using an untargeted MS-based platform for both serum and plasma samples subjected to a post-centrifugation time delay up to 24 h at either 4 or 23 °C. No significant effects were seen with either serum or plasma at either temperature with delays up to 3 h. However, at the next time point, which was 8 h, there were significant effects on metabolite levels at both temperatures in both sample types. Similar effects were seen in plasma samples regardless of whether the blood tube contained a gel separation plug. These findings suggest that limited delays in removal of serum or plasma layers from other blood fractions may be tolerable.

Buffy-Coat Contamination: Plasma can be contaminated by white blood cells if care is not used to avoid getting any of the underlying buffy coat after centrifugation of blood samples. Kamlage et al. [38] assessed two levels of buffy coat contamination and found that, at most, only 3% of metabolite levels were altered, suggesting that the effect was minimal.

### 2.4. Post-Processing

Time Delay and Temperature: Several studies have investigated the consequences of delays at different temperatures between when serum or plasma fractions are made and when they are placed in long-term storage or analyzed. The time delays investigated range from very short (15 min) [37] to days [18,19,29,30] or weeks [41]. The most common temperatures evaluated were 0–4 °C and room temperature. However, one study included an intermediate temperature (12 °C) [38] and three investigated the effect of storage at freezing at a different temperature prior to moving to –80 °C for long-term storage [20,23,41]. 

Short post-processing delays at room temperature of up to 2 h resulted in only minimal changes to metabolite levels measured in serum [37] or plasma [37,38,41] with either NMR [37] or MS-based platforms [38,41]. However, the number of metabolites affected at room temperature increased with time making longer delays unacceptable [19,29,38,41]. Metabolomic profiles and metabolites levels were stable longer at 0–4 °C. Both Anton et al. [29] and Barton et al. [30] observed minimal changes with serum samples for up to 36 h and Moriya et al. [41] reported similar findings for plasma samples at 24 h. The latter study did not test 36 h but found significant changes in about 30% of the metabolites measured after 1 week at 4 °C. The number of metabolites affected by delays at 12 °C for up to 16 h was somewhat more than at 4 °C but less than at room temperature [38]. Finally, to mimic the situation where samples initially frozen at one temperature for some period before transfer to long-term storage, Moriya et al. [41] evaluated the effect of post-processing storage at −30 °C for 1 week or 1 month prior to storage at −80 °C on plasma from five individuals. No metabolites were altered after 1 week whereas <10% were changed after 1 months. In contrast, Pinto et al. [20] found no changes in the plasma metabolome after 1 month at −20 °C with samples from 49 donors. Bervoets et al. [23] evaluated 8 h either on dry ice (−78.5 °C) or in liquid nitrogen (−196 °C) prior to transfer to −80 °C and found no significant effects of these conditions on metabolomic profiles. Overall, these results suggest that very short delays at room temperature are tolerable and the duration of the delay that is acceptable lengthens with lower temperatures.

### 2.5. Storage Conditions

Storage Time: Of the six studies that have assessed the effect of storage time on metabolic profiles, five stored samples at −80 °C [20,23,35,37,48] and one stored the samples in liquid nitrogen [24]. Three of the −80 °C studies with samples numbers ranging from 20 to 96, found no significant effect on the untargeted metabolomic profiles derived using NMR of storage for 3 months [37], 10 months [23], or 30 months [20]. In contrast, using a single pooled sample, Haid et al. [35] found that the levels of 56 of 111 metabolites measured using a targeted MS-based platforms were altered after storage for 5 years. The storage time of this study was double that of the NMR-based studies. However, whether the difference in findings mean that storage time effects only become apparent after 30 months, that they are only detected using more sensitive and quantitative approaches, or if the findings from a study using only one sample are not reliable, is not clear. Yang et al. [48] compared MS-based profiles from plasma stored at −80 °C for 2 months to that stored for 5 years and found numerous metabolites were altered. However, it is not clear that the samples from the two time points were from the same individuals, so differences may reflect more than the effects of storage time. Hebels et al. [24] included longer storage times but compared samples that had been in liquid nitrogen for 13 to 17 years to each other rather than ones at this temperature for much shorter durations. While no significant differences were found in the untargeted MS-based metabolomic profiles of these samples, this result does not inform the question of whether storage time or metabolomic approach and platform influences the findings of these studies. 

Freeze/Thaw Cycles: The effect of freeze/thaw cycles on metabolomic results have been assessed by untargeted NMR studies [9,20,34] and both untargeted [25] and targeted [14,17,29,47] MS-based studies. Although the findings vary somewhat depending on the metabolites measured and criteria used to compare samples, the platform or approach used for the metabolomic analysis did not substantially affect the findings. One or two freeze/thaw cycles were found to non-significantly effect metabolomic profiles from serum [9,14,17,29] and plasma [14,17,20,25,47]. After three freeze/thaw cycles with samples from nine volunteers, Wood et al. [47] reported that the level of one of the 15 metabolites measured changed significantly. Anton et al. [29] observed small, nonsignificant changes in some metabolite levels in 19 samples after four cycles while Pinto et al. [20] noted that changes in the metabolomic profiles of some of the 49 samples used occurred after four cycles. Fliniaux et al. [34] evaluated the effect of either five or 10 freeze/thaw cycles on seven serum samples and found that both the NMR spectroscopic profiles and levels of several metabolites were altered by these treatments. Thus, these findings suggest metabolomic profiles are affected by repeated freeze/thaw cycles in a dose-dependent manner.

### 2.6. Urine

**Figure 3:**
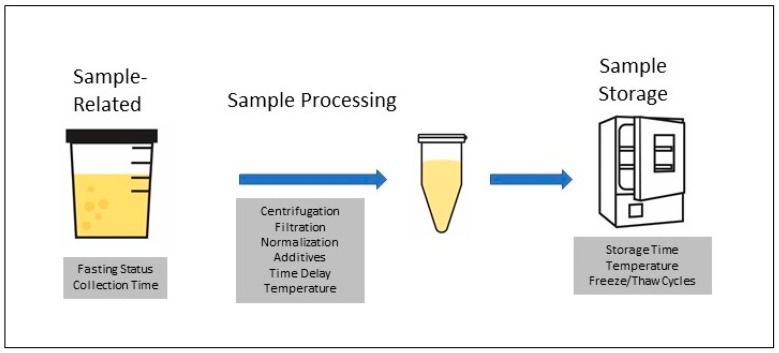
Pre-analytical variables for plasma and serum samples.

The procedure for processing urine samples, which is diagramed in Figure 3, is somewhat more variable than for blood samples. The pre-analytical variables that have been evaluated for their effects of metabolomic profiles as they relate to the urine sample, processing, or storage are listed in the shaded boxes below the relevant step in the procedure. Individual metabolites found to be affected by various conditions in specific studies are listed in Table S1.

Table 2 summarizes the studies of pre-analytical factors that influence metabolomic studies conducted with urine biospecimens and the results are described below.

### 2.7. Urine Sample Collection

Time of Day and Fasting Status: One study has investigated the effect of sample collection at different times of the day and before and after eating a meal on urine metabolomic profiles from an untargeted MS-based platform. Using samples from 26 participants, Kim et al. [39] noted that variability in urine metabolites was somewhat more than for blood. Differences in collection times and fasting status resulted in similar levels of variability in the metabolomic profiles, which was about 8% and was considerably less than the inter-individual or technical variability. These findings led the authors to suggest that the variability from collection time and fasting status should be minimized by controlling for these factors or eliminated by standardizing collection times and requiring fasting samples [39]. 

### 2.8. Urine Processing

Centrifugation Conditions, Filtration, and Additives: Urine samples are processed prior to storage or analysis to remove cellular debris and to minimize the effect of any contaminating bacteria on the metabolomic profiles. In an untargeted MS-based study with urine from two volunteers, Ammerlaan et al. [49], examined the effect of centrifugation time, temperature, rotational speed, and brake force on urine metabolomics. Brake force was the only variable for which a significant difference was found. Of the conditions tested, centrifugation for 20 min at 4 °C at a speed of 12,000× *g* was found to be optimal and resulted in reproducibility in levels of >95% of the metabolites measured.

Methods to minimize bacterial contamination that have been tested are pre-centrifugation, filtration, and treating the urine with additives. Saude and Skyes [50] compared urine samples from two donors stored for 4 weeks at 4 °C or room temperature that were not treated, had been pre-centrifuged, were filtered, or were treated with sodium azide. Pre-centrifugation was somewhat effective at reducing the changes in metabolite levels that occurred at room temperature but filtration with a 0.22 µm syringe filter or treatment with sodium azide was better. Changes in metabolite levels were minimal when samples were maintained at 4 °C. Bernini et al. [19] tested the same variables with six urine samples subjected to a 1-week processing delay and found that either pre-centrifugation at moderate speeds or filtration were effective at removing cells and debris and reduced bacterial contamination. Pre-centrifugation at high speeds appeared to cause cell breakage and altered the metabolomic profile.

Lauridsen at al. [56] assessed changes in the NMR-derived citrate and creatinine resonances of urine samples treated with either sodium azide or sodium fluoride that were stored at 4 °C for up to 1 week. Both additives were effective at inhibiting contamination but some shift in the citrate resonance was observed in the sodium fluoride samples. The addition of phosphate buffer to control the pH of the urine samples was also found to stabilize the NMR signals [56]. Roux et al. [58] compared the NMR-derived metabolomic profiles of a pooled urine sample treated with boric acid to an untreated pooled sample maintained at either room temperature or 4 °C for up to 72 h. Evidence of bacterial contamination was observed after 12–24 h in the untreated samples. This contamination was inhibited at 4 °C and by boric acid. However, boric acid did not block the changes in the metabolomic profiles caused by chemical instability at the longer time points in the room temperature samples. Thus, filtration and treatment with sodium azide appear to be somewhat more effective at inhibiting bacterial contamination of urine samples maintained at room temperature for up to a day after collection. 

Time and Temperature Delays: In separate studies that used 40 urine samples, Barton et al. [30] used an untargeted NMR platform and Dunn et al. [33] used an untargeted MS-based platform to assess the effect of a 24-h delay at 4 °C. Both studies found no evidence of any significant effect on the metabolomic profiles. Budde et al. [51] compared NMR-derived signals from 11 samples subjected to delays of up to 72 h at either room temperature or 10 °C, which was the temperature of the NMR cooling rack. The NMR signals were altered with increased time and temperature but were fairly stable for 24 h at 10 °C. Rotter et al. [57] measured levels of 63 metabolites with a targeted MS-based platform in a pooled urine sample maintained at −20, 4, 9 (cool packs), or 20 °C (room temperature) for 2, 8, or 24 h. A total of 90% of the metabolites were unaffected in these samples. Amino acids were the most sensitive and were altered in the samples kept at either 9 or 20 °C for 24 h. Collectively, these studies suggest that exposure of urine samples to temperatures up to room temperature for short times does not adversely affect the metabolomic profile. However, maintenance of the samples at ≤4 °C should minimize changes to metabolite levels.

Osmolarity and Sample Volume: Unlike blood, where metabolite concentration is maintained through homeostatic regulation, urine concentration can vary significantly from sample to sample. This variation in sample volume can be accounted for by normalizing metabolite levels to some component whose level reflects the concentration, such as creatinine, osmolarity, or specific gravity. Three studies have investigated whether normalization of urine samples before metabolomic analysis may be better than post-analysis normalization of the metabolomic profiles acquired using MS-based platforms [52,53,54]. Edmands et al. [53] used 24 urine samples to compare one pre-acquisition normalization approach to three post-acquisition approaches. Pre-acquisition normalization to specific gravity was found to be superior to post-acquisition normalization to specific gravity, median fold change, or urinary volume for maximizing the total number of MS feature recovered. Chetwynd et al. [52] also found that pre-analysis normalization to osmolarity gave better ability to detect metabolites in five urine samples subjected to serial dilution with water as compared to post-analysis normalization with osmolarity or the mass spectrum total signal. However, the combination of pre- and post-analytic normalization gave the best results. Finally, Gagnebin et al. [54] focused on reducing the variability in the urine metabolomic profile due to sample concentration and found that a sequential strategy of pre-acquisition normalization to osmolarity followed by post-acquisition normalization to MS total useful signal or probabilistic quotient normalization gave the best results. Collectively, these findings suggest that pre-analytical normalization of urine may improve the findings of metabolomic analyses.

### 2.9. Storage Conditions

Storage Time and Temperature: The influence of storage time and temperature on urine metabolomics has been investigated by four studies. Budde et al. [51] found that storage of urine at –80 °C for 1 month resulted in no change in the NMR fingerprint. Similarly, Gika et al. [55] found that storage for 1 month at either −20 or −80 °C did not cause any changes in the MS-derived metabolomic profiles from six samples. Bernini et al. [19] compared urine samples stored for 1 week at −80 °C or in liquid nitrogen that either had or had not been pre-centrifugated prior to freezing. The NMR-derived metabolomic profiles of the samples that were not pre-centrifuged were more affected than the pre-centrifuged samples and the variation was greater in the −80 °C samples than in the liquid nitrogen samples. The difference was suggested to result from cell breakage at –80 °C that was minimized when freezing was more rapid in liquid nitrogen. Finally, Lauridsen et al. [56] found that storage for up to 26 weeks at either −25 or −80 °C resulted in no change in the NMR-derived metabolomic profile. However, freeze drying of the urine followed by rehydration for analysis significantly altered the metabolomic profile. Thus, urine samples appear to be stable for up to 26 weeks when stored at temperatures ≤−25 °C, although the metabolomic profile may be affected if the samples contain cells prior to freezing.

Freeze/Thaw Cycles: In an untargeted MS-based study, Gika et al. [55] found that the metabolomic profiles from urine samples from nine donors were not altered by up to nine freeze-thaw cycles. Rotter et al. [57] evaluated the metabolite levels obtained from 12 urine samples with a targeted MS-based platform. Metabolite levels were unchanged by one or two freeze/thaw cycles, but several were significantly altered after three cycles. Saude and Skyes [50] used a targeted NMR platform and found the levels of many metabolites in two urine samples were changed by eight freeze/thaw cycles. These findings suggest three or more freeze/thaw cycles may affect urine metabolomic results, particularly when targeted approaches are used, and metabolite levels are measured. 

## 3. Discussion

The summary of the findings and conclusions of the 35 studies that examined blood biospecimens and 14 that examined urine biospecimens are presented in Table 3. 

Epidemiological studies often collect samples in nonclinical settings and various types of environments and are subjected to various time and temperature delays prior to being processed and frozen for storage which may affect sensitivity and specificity and reproducibility of results. Therefore, understanding how pre-analytical factors affect metabolite levels is of importance for these studies to either optimize study design or inform about those metabolites that may be affected by collection and processing conditions for study exclusion. 

Several pre-analytical factors appear to have no significant effects on measured metabolites in blood samples including tube additives, oxygenation, centrifugation conditions, and buffy-coat contamination. Hemolysis, which affected the MS-based metabolomic profiles but not those obtained with NMR, was the only pre-analytical factor whose effect varied by platform.

When comparing serum with plasma, plasma seems to tolerate short processing delays better than serum; whereas serum may provide higher sensitivity. Findings regarding which fraction provides better reproducibility are mixed. Therefore, the reviewed studies suggest that both serum and plasma can be used for metabolomics assays; however, they do differ in sensitivity and reliability. We recommend that decisions regarding which biofluid to use should be driven by the study design and hypothesis versus being analytically driven.

Pre- and post-centrifugation and post-processing variables were found to affect blood metabolite stability. Overall, metabolites are more sensitive to delays at room temperature than at 0–4 °C. Pre-centrifugation, delays of just 15 min at room temperature and more than a few hours at 0–4 °C can affect some metabolites. Following centrifugation, delays longer than 3 h at any temperature may affect some metabolites. Finally, during post-processing, most metabolites are unaffected by delays of less than 2 h at room temperature and up to 24 h at 0–4 °C. Thus, the effect of time delay varies depending on the temperature. Implementation of quality assurance and quality control measures is necessary to obtain valid and reproducible results when assaying blood biospecimens for metabolomics studies.

The reviewed studies indicated that metabolites are unaffected by storage of blood samples at −80 °C for up to 30 months. However, epidemiological studies have archived samples for many decades. For example, blood samples from the Framingham Heart Study’s (FHS) original cohort were collected in 1948, while the second-generation (Offspring cohort) samples were collected in 1971 and the Third-Generation cohort in 2002 [59]. Over the past decade, the FHS cohorts have undergone omics profiling, including metabolomics [59]. Thus, studies of the effects of blood storage are needed to better assess the impact of long-term storage on archival samples. Additionally, with these studies, the samples are used multiple times for various studies, resulting in multiple freeze/thaw cycles. Evidence suggests that these multiple freeze/thaw cycles alter many metabolites and therefore, minimizing repeated freezing and thawing of samples is recommended. 

Other factors to consider during the study design process are fasting status and when the samples are collected, as both the time of day and season can affect some metabolites. Most metabolites appear to be unaffected by fasting status except for amino acids, hexoses, and some lipids which were minimally affected. Therefore, fasting status should be considered in the study design if one is examining one of the metabolite classes affected. If one is using archival samples where subjects did not fast, those metabolites affected by fasting status can be identified and removed from the analysis allowing for the samples to be used.

Collection time of day only affected specific metabolites, particularly bile acids and vitamin metabolites. Metabolites affected by season of blood collection are bile acids, organic acids, purines and pyrimidines. Thus, these factors should be considered during the study design phase, when possible, if the metabolites of interest are affected by collection time of day or season. If using archival samples, this information should be considered in the analysis phase.

For urine, there are fewer studies to draw conclusions from; however, a few key points can be made. Metabolites were unaffected by variation in centrifugation temperatures and time and centrifugation may be useful for removing bacterial and cellular debris. Centrifugation speed also has no effect except for very high speed, which may cause cellular breakage and contamination with cellular metabolites. Furthermore, filtration and treatment with sodium azide reduce bacterial contamination of samples. 

Urine sample concentration can vary significantly from sample to sample; therefore, normalization of metabolite levels is necessary. The results from the three studies examining normalization collectively suggest that pre-analytical normalization may be preferred to post-analytic normalization.

When examining time and temperature delays when processing urine, metabolites are unaffected by short delays at room temperature and longer delays at 0–4 °C. However, in one study amino acids were altered if kept at either 9 or 20 °C for 24 h; therefore, maintaining samples at ≤4 °C should minimize the effect on metabolite levels. 

Similar to the blood samples, urine samples are archived for decades after collection. The results of the four studies examining storage time and temperature show that metabolites are unaffected by storage at <−25 °C for up to 26 months. However, studies of longer storage times are needed to better determine the impact of long-term storage on archival samples. Equally important are the effects of repeated freezing and thawing of urine samples. Three studies examined freeze/thaw cycles with mixed results; however, these studies suggest that three or more freeze thaws may affect metabolite levels, particularly when an untargeted approach is used. 

Only one study investigated fasting status and the collection time of day for urine. Differences in collection times and fasting status resulted in some variability in metabolite levels suggesting that standardized collection times and requiring fasting may minimize these effects. Additional studies may be required to further examine these variables.

This summary of the literature examining pre-analytical factors provides the first compilation of relevant data and offers insight into possible considerations for quality assurance and quality control measure implementation.

## 4. Materials and Methods 

### 4.1. Search Strategy and Selection Criteria

We conducted a search in PubMed and EMBASE from inception through 14 January 2019 on articles that examined best practices for metabolomic sample handling. Search terms used were the MeSH terms: “metabolomics” or “metabolome” or “metabolomic” in combination with the keywords “pre-analytical,” “preanalytical,” “pre-analytics,” “preanalytics,” “stability,” “reproducibility of results,” “variability,” “processing delay,” “delayed processing,” “freezing delay,” or “delayed freezing.” The search was restricted to articles written in English. A manual search was also used to investigate papers referenced by included papers as well as to identify relevant studies that did not include the MeSH terms in either their title or abstract or were in journals not indexed by PubMed or EMBASE. 

Studies were eligible for inclusion if they met the following criteria: studies had to have been conducted in human serum, plasma, or urine; metabolomics analysis had to include more than one chemical class; and at least one pre-analytical condition was investigated. Papers were excluded if they were reviews, did not use samples from humans, used whole blood or red blood cells, cerebrospinal fluid, or specific tissues, or examined only factors related to the participants (other than fasting status) or only tested reproducibility over time. Two reviewers independently assessed all titles and abstracts. 

### 4.2. Data Extraction

A standardized protocol was used for extracting data from full-text articles on the following characteristics: sample type (plasma, serum and urine), platform utilized (nuclear magnetic resonance (NMR) or mass spectrometry (MS)-based), targeted/untargeted approach, sample-related variables, sample processing-related variables, post-processing variables, and sample storage. The studies included, and these characteristics, are listed in Table 1 (for blood studies) and Table 2 (for urine studies).

## Figures and Tables

**Table 1:** Studies of pre-analytical factors that influence metabolomic studies conducted with blood.

Publication	Blood Fraction	Platform	Approach	Sample-Related	Sample Processing	Post-Processing	Sample Storage
Ammerlaan 2014 [27]	Serum and Plasma	MS-Based	Untargeted		Centrifugation Conditions		
Ang 2012 [28]	Plasma	MS-Based	Untargeted	Collection Time			
Anton 2015 [29]	Serum	MS-Based	Targeted			Time Delay Temperature	Freeze/Thaw Cycles
Barton 2008 [30]	Serum	NMR	Untargeted			Time Delay	
Bernini 2011 [19]	Serum and Plasma	NMR	Untargeted		Time Delay (PreC) Temperature (PreC)	Time Delay	
Bervoets 2015 [23]	Plasma	NMR	Untargeted	Hemolysis Oxygenation Tube Additives	Centrifugation Conditions Time Delay (PreC) Temperature (PreC)	Temperature	Storage time
Breier 2014 [14]	Serum and Plasma	MS-Based	Targeted	Tube Additives	Time Delay (PreC) Temperature (PreC)		Freeze/Thaw Cycles
Brunius 2017 [31]	Plasma	NMR	Untargeted		Time Delay (PreC) Temperature (PreC)		
Carayol 2015 [32]	Serum	MS-Based	Targeted	Fasting Status			
Denery 2011 [15]	Serum and Plasma	MS-Based	Untargeted	Serum vs. Plasma Tube Additives			
Dunn 2008 [33]	Serum	MS-Based	Untargeted		Time Delay (PreC)		
Fliniaux 2011 [34]	Serum	NMR	Untargeted		Time Delay (PreC) Temperature (PreC)		Freeze/Thaw Cycles
Haid 2018 [35]	Plasma	MS-Based	Targeted				Storage time
Hebels 2013 [24]	Plasma	MS-Based	Untargeted	Tube Additives	Time Delay (PreC)		Storage Time
Hirayama 2015 [17]	Serum and Plasma	MS-Based	Targeted		Time Delay (PreC)	Time Delay Temperature	Freeze/Thaw Cycles
Jain 2017 [36]	Plasma	MS-Based	Untargeted		Time Delay (PreC)		
Jobard 2016 [37]	Serum and Plasma	NMR	Untargeted		Centrifugation Conditions Time Delay (PreC) Temperature (PreC)	Time Delay	Storage time
Kamlage 2014 [38]	Plasma	MS-Based	Untargeted and Targeted	Hemolysis	Time Delay (PreC) Temperature (PreC) Buffy Coat Contamination	Time Delay Temperature	
Kamlage 2018 [18]	Serum and Plasma	MS-Based	Untargeted		Time Delay (PreC)	Time Delay	
Kim 2014 [39]	Plasma	MS-Based	Untargeted	Collection Time Fasting Status			
Lesche 2016 [40]	Plasma	NMR and MS-Based	Untargeted		Centrifugation Conditions		
Malm 2016 [26]	Serum and Plasma	MS-Based	Untargeted	Tube Additives	Time Delay (PreC, PreS) Temperature (PreC, PreS)		
Midttun 2014 [22]	Plasma	MS-Based	Targeted	Tube Additives	Time Delay (PreC)		
Moriya 2016 [41]	Plasma	MS-Based	Untargeted			Time Delay Temperature	
Nishiumi 2018 [10]	Serum and Plasma	MS-Based	Untargeted	Serum vs. Plasma	Time Delay (PreC) Temperature (PreC)		
Paglia 2018 [11]	Serum and Plasma	MS-Based	Targeted	Serum vs. Plasma Tube Additives			
Pinto 2014 [20]	Plasma	NMR	Untargeted	Tube additives Fasting		Temperature	Freeze/Thaw Cycles Storage Time
Sampson 2013 [42]	Serum and Plasma	MS-Based	Targeted	Fasting status			
Teahan 2006 [9]	Serum and Plasma	NMR	Untargeted	Serum vs. Plasma	Time Delay (PreC) Temperature (PreC)		Freeze/Thaw Cycles
Thompson 2012 [43]	Serum	MS-Based	Targeted	Fasting Status			
Townsend 2013 [21]	Plasma	MS-Based	Untargeted	Fasting Status Tube Additives	Time Delay (PreC)		
Townsend 2016 [44]	Plasma	MS-Based	Untargeted	Fasting Status Collection Season Collection Time			
Trezzi 2016 [45]	Plasma	MS-Based	Targeted		Time Delay (PreC) Temperature (PreC)		
Wang 2018 [46]	Plasma	MS-Based	Untargeted		Time Delay (PreC)		
Wedge 2011 [12]	Serum and Plasma	MS-Based	Untargeted	Serum vs. Plasma			
Wood 2008 [47]	Plasma	MS-Based	Targeted		Time Delay (PreC)		Freeze/Thaw Cycles
Yang 2013 [48]	Plasma	MS-Based	Untargeted				Storage Time
Yin 2013 [25]	Serum and Plasma	MS-Based	Untargeted	Tube Additives Hemolysis	Time Delay (PreC) Temperature (PreC)		Freeze/Thaw Cycles
Yu 2011 [13]	Plasma and Serum	MS-Based	Targeted	Serum vs. Plasma			

PreC, pre-centrifugation; PreS, pre-separation.

**Table 2:** Studies of pre-analytical factors that influence metabolomic studies conducted with urine.

Publication	Platform	Approach	Sample-Related	Sample Processing	Sample Storage
Ammerlaan 2014 [49]	MS-Based	Untargeted		Centrifugation Conditions	
Barton 2008 [30]	NMR	Untargeted		Time Delay	
Bernini 2011 [19]	NMR	Untargeted		Additives Centrifugation Conditions Filtration	Storage Time Storage Temperature
Budde 2016 [51]	NMR	Targeted		Time Delay Temperature	Storage Time
Chetwynd 2016 [52]	MS-Based	Untargeted		Osmolarity and Sample Volume	
Dunn 2008 [33]	MS-Based	Untargeted		Time Delay	
Edmands 2014 [53]	MS-Based	Untargeted		Osmolarity and Sample Volume	
Gagnebin 2017 [54]	MS-Based	Untargeted		Osmolarity and Sample Volume	
Gika 2007 [55]	MS-Based	Untargeted			Storage Time Storage Temperature Freeze/Thaw Cycles
Kim 2014 [39]	MS-Based	Untargeted	Collection Time Fasting Status		
Lauridsen 2007 [56]	NMR	Untargeted		Additives	Storage Temperature Storage Time
Rotter 2017 [57]	MS-Based	Targeted		Time Delay Temperature	Freeze/Thaw Cycles
Roux 2015 [58]	NMR and MS-Based	Targeted		Additives Time Delay Temperature	
Saude and Skyes 2007 [50]	NMR	Targeted		Centrifugation Conditions Filtration Additives	Storage Times Storage Temperature Freeze/Thaw Cycles

**Table 3:** Summary of findings. Bold text indicates that the pre-analytical factor may affect metabolomic results.

Analytic Factor	Summary of Findings/Conclusions
**Blood Samples**	
**Serum vs. plasma**	**Plasma samples appear to tolerate short processing delays better than serum samples.** **Serum samples may provide higher sensitivity.** **Findings regarding which fraction provides better reproducibility are mixed.**
Tube additives	Does not significantly affect metabolomic profiles.
**Fasting status**	**Some metabolites may be affected.**
**Hemolysis**	**MS-based analyses may be affected by hemolysis.**
Oxygenation	Does not significantly affect metabolomic profiles.
**Collection time of day and season**	**These variables may affect some metabolites.**
**Pre-centrifugation time delay and temperature**	**Metabolites are more sensitive to delays at room temperature than at 0–4 °C. Room temperature delays of any length and more than a few hours at 0–4 °C affect some metabolites.**
Centrifugation conditions	Does not significantly affect metabolomic profiles.
**Post-centrifugation time delay and temperature**	**Delays longer than 3 h at any temperature may affect some metabolites.**
Buffy-coat contamination	Does not significantly affect metabolomic profiles.
**Post-processing time delay and temperature**	**Most metabolites are unaffected by delays of <2 h at room temperature and up to 24 h at 0–4 °C.**
**Storage time**	**Metabolites are unaffected by storage at −80 °C for up to 30 months. Studies of longer storage times are needed.**
**Freeze/thaw cycles**	**Multiple freeze/thaw cycles alter many metabolites.**
**Urine Samples**	
**Collection time and fasting**	**These variables may affect some metabolites.**
**Centrifugation conditions**	**Pre-centrifugation may be useful for removing bacterial and cellular debris. Metabolites were unaffected by variation in speed, temperatures and time.**
**Filtration and additives**	**Filtration and treatment with sodium azide reduce bacterial contamination of samples.**
Time delay and temperature	Metabolites are unaffected by short delays at room temperature and longer delays at 0–4 °C.
**Osmolarity and Sample Volume**	**Pre-analytical normalization may be better than post-analytic normalization.**
Storage time and temperature	Metabolites are unaffected by storage at <−25 °C for up to 26 months. Studies of longer storage times are needed.
**Freeze/thaw cycles**	**Results are mixed but multiple freeze/thaw cycles may affect some metabolites.**

## Supplementary Materials

The following are available online at https://www.mdpi.com/2218-1989/9/8/156/s1, Table S1: Metabolites affected by pre-analytical factors.

## References

[1] Rattray N.J.W., Deziel N.C., Wallach J.D., Khan S.A., Vasiliou V., Ioannidis J.P.A., Johnson C.H. (2018). Beyond genomics: understanding exposotypes through metabolomics. Hum. Genom..

[2] Erben V., Bhardwaj M., Schrotz-King P., Brenner H. (2018). Metabolomics biomarkers for detection of colorectal neoplasms: A systematic review. Cancers.

[3] Newgard C.B. (2017). Metabolomics and metabolic diseases: Where do we stand?. Cell Metab..

[4] Ruiz-Canela M., Hruby A., Clish C.B., Liang L., Martinez-Gonzalez M.A., Hu F.B. (2017). Comprehensive metabolomic profiling and incident cardiovascular disease: A systematic review. J. Am. Heart Assoc..

[5] Yin P., Lehmann R., Xu G. (2015). Effects of pre-analytical processes on blood samples used in metabolomics studies. Anal. Bioanal. Chem..

[6] Hernandes V.V., Barbas C., Dudzik D. (2017). A review of blood sample handling and pre-processing for metabolomics studies. Electrophoresis.

[7] Dudzik D., Barbas-Bernardos C., Garcia A., Barbas C. (2018). Quality assurance procedures for mass spectrometry untargeted metabolomics. A review. J. Pharm. Biomed. Anal..

[8] Kirwan J.A., Brennan L., Broadhurst D., Fiehn O., Cascante M., Dunn W.B., Schmidt M.A., Velagapudi V. (2018). Preanalytical processing and biobanking procedures of biological samples for metabolomics research: A white paper, community perspective (for “Precision medicine and pharmacometabolomics task group”-The metabolomics society initiative). Clin. Chem.

[9] Teahan O., Gamble S., Holmes E., Waxman J., Nicholson J.K., Bevan C., Keun H.C. (2006). Impact of analytical bias in metabonomic studies of human blood serum and plasma. Anal. Chem..

[10] Nishiumi S., Suzuki M., Kobayashi T., Yoshida M. (2018). Differences in metabolite profiles caused by pre-analytical blood processing procedures. J. Biosci. Bioeng..

[11] Paglia G., Del Greco F.M., Sigurdsson B.B., Rainer J., Volani C., Hicks A.A., Pramstaller P.P., Smarason S.V. (2018). Influence of collection tubes during quantitative targeted metabolomics studies in human blood samples. Clin. Chim. Acta.

[12] Wedge D.C., Allwood J.W., Dunn W., Vaughan A.A., Simpson K., Brown M., Priest L., Blackhall F.H., Whetton A.D., Dive C. (2011). Is serum or plasma more appropriate for intersubject comparisons in metabolomic studies? An assessment in patients with small-cell lung cancer. Anal. Chem..

[13] Yu Z., Kastenmuller G., He Y., Belcredi P., Moller G., Prehn C., Mendes J., Wahl S., Roemisch-Margl W., Ceglarek U. (2011). Differences between human plasma and serum metabolite profiles. PLoS ONE.

[14] Breier M., Wahl S., Prehn C., Fugmann M., Ferrari U., Weise M., Banning F., Seissler J., Grallert H., Adamski J. (2014). Targeted metabolomics identifies reliable and stable metabolites in human serum and plasma samples. PLoS ONE.

[15] Denery J.R., Nunes A.A., Dickerson T.J. (2011). Characterization of differences between blood sample matrices in untargeted metabolomics. Anal. Chem..

[16] Benini Z.L., Camilloni M.A., Scordato C., Lezzi G., Savia G., Oriani G., Bertoli S., Balzola F., Liuzzi A., Petroni M.L. (2001). Contribution of weight cycling to serum leptin in human obesity. Int. J. Obes..

[17] Hirayama A., Sugimoto M., Suzuki A., Hatakeyama Y., Enomoto A., Harada S., Soga T., Tomita M., Takebayashi T. (2015). Effects of processing and storage conditions on charged metabolomic profiles in blood. Electrophoresis.

[18] Kamlage B., Neuber S., Bethan B., Gonzalez Maldonado S., Wagner-Golbs A., Peter E., Schmitz O., Schatz P. (2018). Impact of prolonged blood incubation and extended serum storage at room temperature on the human serum metabolome. Metabolites.

[19] Bernini P., Bertini I., Luchinat C., Nincheri P., Staderini S., Turano P. (2011). Standard operating procedures for pre-analytical handling of blood and urine for metabolomic studies and biobanks. J. Biomol. NMR.

[20] Pinto J., Domingues M.R., Galhano E., Pita C., Almeida Mdo C., Carreira I.M., Gil A.M. (2014). Human plasma stability during handling and storage: impact on NMR metabolomics. Analyst.

[21] Townsend M.K., Clish C.B., Kraft P., Wu C., Souza A.L., Deik A.A., Tworoger S.S., Wolpin B.M. (2013). Reproducibility of metabolomic profiles among men and women in 2 large cohort studies. Clin. Chem..

[22] Midttun O., Townsend M.K., Nygard O., Tworoger S.S., Brennan P., Johansson M., Ueland P.M. (2014). Most blood biomarkers related to vitamin status, one-carbon metabolism, and the kynurenine pathway show adequate preanalytical stability and within-person reproducibility to allow assessment of exposure or nutritional status in healthy women and cardiovascular patients. J. Nutr..

[23] Bervoets L., Louis E., Reekmans G., Mesotten L., Thomeer M., Adriaensens P., Linsen L. (2015). Influence of preanalytical sampling conditions on the ^1^H NMR metabolic profile of human blood plasma and introduction of the Standard PREanalytical Code used in biobanking. Metabolomics.

[24] Hebels D.G., Georgiadis P., Keun H.C., Athersuch T.J., Vineis P., Vermeulen R., Portengen L., Bergdahl I.A., Hallmans G., Palli D. (2013). Performance in omics analyses of blood samples in long-term storage: opportunities for the exploitation of existing biobanks in environmental health research. Environ. Health Perspect..

[25] Yin P., Peter A., Franken H., Zhao X., Neukamm S.S., Rosenbaum L., Lucio M., Zell A., Haring H.U., Xu G. (2013). Preanalytical aspects and sample quality assessment in metabolomics studies of human blood. Clin. Chem..

[26] Malm L., Tybring G., Moritz T., Landin B., Galli J. (2016). Metabolomic quality assessment of EDTA plasma and serum samples. Biopreserv. Biobank..

[27] Ammerlaan W., Trezzi J.P., Lescuyer P., Mathay C., Hiller K., Betsou F. (2014). Method validation for preparing serum and plasma samples from human blood for downstream proteomic, metabolomic, and circulating nucleic acid-based applications. Biopreserv. Biobank..

[28] Ang J.E., Revell V., Mann A., Mantele S., Otway D.T., Johnston J.D., Thumser A.E., Skene D.J., Raynaud F. (2012). Identification of human plasma metabolites exhibiting time-of-day variation using an untargeted liquid chromatography-mass spectrometry metabolomic approach. Chronobiol. Int..

[29] Anton G., Wilson R., Yu Z.H., Prehn C., Zukunft S., Adamski J., Heier M., Meisinger C., Romisch-Margl W., Wang-Sattler R. (2015). Pre-analytical sample quality: metabolite ratios as an intrinsic marker for prolonged room temperature exposure of serum samples. PLoS ONE.

[30] Barton R.H., Nicholson J.K., Elliott P., Holmes E. (2008). High-throughput 1H NMR-based metabolic analysis of human serum and urine for large-scale epidemiological studies: validation study. Int. J. Epidemiol..

[31] Brunius C., Pedersen A., Malmodin D., Karlsson B.G., Andersson L.I., Tybring G., Landberg R. (2017). Prediction and modeling of pre-analytical sampling errors as a strategy to improve plasma NMR metabolomics data. Bioinformatics.

[32] Carayol M., Licaj I., Achaintre D., Sacerdote C., Vineis P., Key T.J., Onland Moret N.C., Scalbert A., Rinaldi S., Ferrari P. (2015). Reliability of serum metabolites over a two-year period: A targeted metabolomic approach in fasting and non-fasting samples from EPIC. PLoS ONE.

[33] Dunn W.B., Broadhurst D., Ellis D.I., Brown M., Halsall A., O’Hagan S., Spasic I., Tseng A., Kell D.B. (2008). A GC-TOF-MS study of the stability of serum and urine metabolomes during the UK Biobank sample collection and preparation protocols. Int. J. Epidemiol..

[34] Fliniaux O., Gaillard G., Lion A., Cailleu D., Mesnard F., Betsou F. (2011). Influence of common preanalytical variations on the metabolic profile of serum samples in biobanks. J. Biomol. NMR.

[35] Haid M., Muschet C., Wahl S., Romisch-Margl W., Prehn C., Moller G., Adamski J. (2018). Long-term stability of human plasma metabolites during storage at −80 degrees C. J. Proteome Res..

[36] Jain M., Kennedy A.D., Elsea S.H., Miller M.J. (2017). Analytes related to erythrocyte metabolism are reliable biomarkers for preanalytical error due to delayed plasma processing in metabolomics studies. Clin. Chim. Acta.

[37] Jobard E., Tredan O., Postoly D., Andre F., Martin A.L., Elena-Herrmann B., Boyault S. (2016). A systematic evaluation of blood serum and plasma pre-analytics for metabolomics cohort studies. Int. J. Mol. Sci..

[38] Kamlage B., Maldonado S.G., Bethan B., Peter E., Schmitz O., Liebenberg V., Schatz P. (2014). Quality markers addressing preanalytical variations of blood and plasma processing identified by broad and targeted metabolite profiling. Clin. Chem..

[39] Kim K., Mall C., Taylor S.L., Hitchcock S., Zhang C., Wettersten H.I., Jones A.D., Chapman A., Weiss R.H. (2014). Mealtime, temporal, and daily variability of the human urinary and plasma metabolomes in a tightly controlled environment. PLoS ONE.

[40] Lesche D., Geyer R., Lienhard D., Nakas C.T., Diserens G., Vermathen P., Leichtle A.B. (2016). Does centrifugation matter? Centrifugal force and spinning time alter the plasma metabolome. Metabolomics.

[41] Moriya T., Satomi Y., Kobayashi H. (2016). Intensive determination of storage condition effects on human plasam metabolomics. Metabolomics.

[42] Sampson J.N., Boca S.M., Shu X.O., Stolzenberg-Solomon R.Z., Matthews C.E., Hsing A.W., Tan Y.T., Ji B.T., Chow W.H., Cai Q. (2013). Metabolomics in epidemiology: sources of variability in metabolite measurements and implications. Cancer Epidemiol. Biomark. Prev..

[43] Thompson D.K., Sloane R., Bain J.R., Stevens R.D., Newgard C.B., Pieper C.F., Kraus V.B. (2012). Daily variation of serum acylcarnitines and amino acids. Metabolomics.

[44] Townsend M.K., Bao Y., Poole E.M., Bertrand K.A., Kraft P., Wolpin B.M., Clish C.B., Tworoger S.S. (2016). Impact of pre-analytic blood sample collection factors on metabolomics. Cancer Epidemiol. Biomarkers Prev..

[45] Trezzi J.P., Bulla A., Bellora C., Rose M., Lescuyer P., Kiehntopf M., Hiller K., Betsou F. (2016). LacaScore: A novel plasma sample quality control tool based on ascorbic acid and lactic acid. Metabolomics.

[46] Wang Y., Carter B.D., Gapstur S.M., McCullough M.L., Gaudet M.M., Stevens V.L. (2018). Reproducibility of non-fasting plasma metabolomics measurements across processing delays. Metabolomics.

[47] Wood J.T., Williams J.S., Pandarinathan L., Courville A., Keplinger M.R., Janero D.R., Vouros P., Makriyannis A., Lammi-Keefe C.J. (2008). Comprehensive profiling of the human circulating endocannabinoid metabolome: clinical sampling and sample storage parameters. Clin. Chem. Lab. Med..

[48] Yang W., Chen Y., Xi C., Zhang R., Song Y., Zhan Q., Bi X., Abliz Z. (2013). Liquid chromatography-tandem mass spectrometry-based plasma metabonomics delineate the effect of metabolites’ stability on reliability of potential biomarkers. Anal. Chem..

[49] Ammerlaan W., Trezzi J.P., Mathay C., Hiller K., Betsou F. (2014). Method validation for preparing urine samples for downstream proteomic and metabolomic applications. Biopreserv. Biobank..

[50] Saude E.J., Sykes B.D. (2007). Urine stability for metabolomic studies: effects of preparation and storage. Metabolomics.

[51] Budde K., Gok O.N., Pietzner M., Meisinger C., Leitzmann M., Nauck M., Kottgen A., Friedrich N. (2016). Quality assurance in the pre-analytical phase of human urine samples by (1)H NMR spectroscopy. Arch. Biochem. Biophys..

[52] Chetwynd A.J., Abdul-Sada A., Holt S.G., Hill E.M. (2016). Use of a pre-analysis osmolality normalisation method to correct for variable urine concentrations and for improved metabolomic analyses. J. Chromatogr. A.

[53] Edmands W.M., Ferrari P., Scalbert A. (2014). Normalization to specific gravity prior to analysis improves information recovery from high resolution mass spectrometry metabolomic profiles of human urine. Anal. Chem..

[54] Gagnebin Y., Tonoli D., Lescuyer P., Ponte B., de Seigneux S., Martin P.Y., Schappler J., Boccard J., Rudaz S. (2017). Metabolomic analysis of urine samples by UHPLC-QTOF-MS: Impact of normalization strategies. Anal. Chim. Acta.

[55] Gika H.G., Theodoridis G.A., Wingate J.E., Wilson I.D. (2007). Within-day reproducibility of an HPLC-MS-based method for metabonomic analysis: Application to human urine. J. Proteome Res..

[56] Lauridsen M., Hansen S.H., Jaroszewski J.W., Cornett C. (2007). Human urine as test material in 1H NMR-based metabonomics: recommendations for sample preparation and storage. Anal. Chem..

[57] Rotter M., Brandmaier S., Prehn C., Adam J., Sylvia R., Gawrych K., Bruning T., Illig T., Lickert H., Adamski J. (2017). Stability of targeted metabolite profiles of urine samples under different storage conditions. Metabolomics.

[58] Roux A., Thevenot E.A., Seguin F., Olivier M.-F., Junot C. (2015). Impact of collection conditions on the metabolite content of human urine samples as analyzed by liquid chromatography coupled to mass spectrometry and nuclear magnetic resonance spectroscopy. Metabolomics.

[59] Andersson C., Johnson A.D., Benjamin E.J., Levy D., Vasan R.S. (2019). 70-year legacy of the Framingham Heart Study. Nat. Rev. Cardiol..

