# Supplementary material for: Pre-Analytical Factors that Affect Metabolite Stability in Human Urine, Plasma, and Serum: A Review
Source: Metabolites. 2019 Jul 25;9(8):156. doi: 10.3390/metabo9080156 (PMC6724180; doi:10.3390/metabo9080156)
Supplement: Supplementary file 1 [file metabolites-09-00156-s001.pdf]

Table S1. Metabolites affected by pre-analytical factors.

Analytic factor	Ref	Affected Metabolites
		Blood Samples
Serum vs. plasma	[1]	Higher in serum than plasma: charged peptides, peptide fragments; higher in plasma than serum: lysoPI.
	[2]	Levels varied between serum and plasma: carbohydrates, organic acids, amino acids and lipids. Details can be found in Nishiumi et al., Figure 2.
	[3]	Higher in serum than plasma: glutamine acid, arginine, taurine; higher in EDTA plasma vs serum: sarcosine. Details can be found in Paglia et al., Table 1 and Figures 3 and 4.
	[4]	Found in serum not plasma: sulfate, dipropylacetic acid, leucine, serine, methionine, arachidonic acid, α-glucopyranosiduronic acid, citrate, cellobiose, eicosanoic acid; found in plasma not serum: 2-methylsuccinic acid, α-hydroxybutyric acid, aspartic acid, 4-hydroxyproline, 3-phenylpropanoic acid, tyrosine, α-linolenic acid, urea, citrulline.
	[5]	Higher in serum than plasma: arginine, PC (C38:1), lysoPC (C16:0), lysoPC (C17:0), lysoPC (C18:0), lysoPC (C18:1), serine, phenylalanine, and glycine.
Tube additives	[6]	Methionine sulfoxide higher in serum from a gel-barrier tube than for tube with clot activator.
	[7]	Variability greater for bile acids and carbohydrates in heparin plasma than in EDTA plasma. Details can be found in Townsend et al., Table 3.
	[3]	Higher in citrate plasma than EDTA plasma: glutamic acid; higher in EDTA plasma than citrate plasma: sarcosine. Details can be found in Paglia et al., Table 1 and Figures 3 and 4.
Fasting status	[8]	Fasting status affected taurine, acylcarnitines, PCs and sphingolipids. Details can be found in Carayol et al., Supplementary Table S1.
	[7]	Fasting status affected bile acids, purines, pyrimidines, and vitamins.
	[9]	Fasting status affected bile acids and vitamins.
Hemolysis	[10]	Metabolites affected include signaling metabolites, amino acids, lipids, carbohydrates, energy metabolites, hormones, vitamins, cofactors, nucleobases, and miscellaneous. Details can be found in Kamlage et al., Table S1.
	[11]	Hemolysis increased levels of hemoglobin and lysoPCs.
Collection time of day and season	[12]	Time of day of blood collection affected acylcarnitines, lysophospholipids, bilirubin, corticosteroids, and amino acids. Details can be found in Ang et al., Table 1.
	[9]	Time of day of blood collection affected bile acids and vitamins.
	[9]	Season of blood collection affected bile acids, purines, pyrimidines and organic acids.
Pre-centrifugation time delay and temperature	[13]	Delayed processing for up to 24 h at either 4^0C or 25^0C affected glucose, pyruvate, and lactate.
	[14]	Delayed for up to 8 h at 4^0C for up to 8 h affected lactate, pyruvate, and glucose.
	[6]	Delays for up to 24 h on cold packs affected arginine, methionine, serotonin, hexose and other metabolites. Details can be found in Breier et al., Figures S3-S12.
	[15]	Delays of up to 36 h at either 4^0C or 22^0C affected pyruvate, lactate and ornithine. Details can be found in Brunius et al., Supplementary Table S1 and Figure S3
	[16]	Delays of up to 24 h at either 4^0C or room temperature affected lactate and glucose.
	[17]	Delays of up to 6 h at room temperature affected aspartate, glutamate, and methionine.
	[18]	Delays of up to 20 h at room temperature affected metabolites involved in glycolysis/gluconeogenesis/pyruvate metabolism, phospholipid and purine metabolism, the TCA cycle, fatty acids, amino acids and other metabolites. Details can be found in Jain et al., Figures 1-3 and Table 2.
	[19]	Delays of up to 6 h at 4^0C or 22^0C affected lactate, glucose, fatty acids, choline and acetone. Details can be found in Jobard et al., Table S1.

	[10]	Delays of up to 2 h at room temperature or up to 6 h on wet ice affected carbohydrates and lipids. Details can be found in Kamalage et al. 2014, Table 3.
	[20]	Delays of up to 6 h at room temperature affected amino acids, carbohydrates, energy metabolism metabolites, nucleobases, vitamins, cofactors, and other metabolites. Details can be found in Kamlage et al. 2018 Table 1, Figures 2, 3, 4, and 6, and Supplemental Tables S1 and S2.
	[21]	Delays up to 24 h at either 4⁰C or 23⁰C affected amino acids, carboxylic acids, fatty acids, fatty alcohols, monosaccharides, sugar acids, inositol, palmitic acid, lactic acid, uric acid, α-ketoglutaric acid, creatinine, adenosine-5-monophosphate, cholesterol, taurine, and urea. Details can be found in Malm et al., Figure 2.
	[22]	Delays up 48 h on ice affected B vitamins, amino acids, kynurenines, other metabolites. Details can be found in Midttun et al., Figure 1.
	[2]	Delays of up to 30 minutes at room temperature or up to 8 h at cold temperatures affected kynurenine, pyruvate, and organic acids. Details can be found in Nishiumi et al., Figures 3 and S1.
	[23]	Delays of up to 23 h at either 4⁰C or room temperature affected lactic acid and ascorbic acid.
	[7]	Delays of up to 48 h on ice packs affected carbohydrates, purines, and pyrimidines.
	[24]	Delays of up to 48 h at 4⁰C affected nucleotides. Details can be found in Wang et al., Figure 1.
	[25]	Delays of up to 8 h at 4⁰C affected oleoylethanolamine, anandamide, palmitoylethanolamine, and docosahexaenoylethanolamine.
	[11]	Delays of up to 24 h at room temperature affected hypoxanthine, sphingosine-1-phosphate, and linolenyl carnitine.
Centrifugation conditions	[26]	Centrifugation speed affected glycerophosphocholines and sphingomyelins.
Post-centrifugation time delay and temperature	[21]	Delays up to 24 h at either 4⁰C or 23⁰C before separation of plasma affected amino acids, carboxylic acids, fatty acids, fatty alcohols, monosaccharides, sugar acids, inositol, palmitic acid, lactic acid, uric acid, α-ketoglutaric acid, creatinine, adenosine-5-monophosphate, cholesterol, taurine, and urea. Details can be found in Malm et al., Figure 2.
Buffy-coat contamination	[10]	Contamination with buffy coat affected inositols and lipids. Details can be found in Kamlage et al. 2014, Table S1.
Post-processing time delay and temperature	[27]	Post-processing delays of up to 36 h at room temperature affected acylcarnitines, amino acids, lyosPCs and PCs. Details can be found in Anton et al., Table 1.
	[28]	Post-processing delays of up to 36 h at 4⁰C affected trimethylamine-N-oxide (TMAO).
	[13]	Post-processing delays of up to 24 h at room temperature affected albumin, triglycerides, LDL/VLDL, proline, citrate and histidine.
	[17]	Post-processing delays of up to 60 minutes at room temperature affected tyrosine, serine, phenylalanine, aspartic acid, isoleucine, glutamate, methionine, and cysteine. Details can be found in Hirayama et al., Supplementary Table S3.
	[10]	Post-processing delays of up to 16 h at 4⁰C, 12⁰C, or room temperature affected signaling metabolites and amino acids. Details can be found in Kamlage et al. 2014, Table 2.
	[20]	Post-processing delays of up to 24 h at room temperature affected ribose, aspartate, and phenylalanine.
	[29]	Post-processing delays of up 24 h at room temperature or 1 week at 4⁰C affected eicosapentaenoate, 2-stearoylglycerophosphocholine, glycerophosphocholine, other lipids, cysteine, Y-glutamyl amino acids, 5-ocoproline, N^6-methyladenosine and allantoin. Details can be found in Moriya et al., Supplemental Figure 2.
Storage time	[30]	Storage for up to 5 years at -80⁰C affected amino acids, hexoses, acylcarnitines and lipids. Details can be found in Haid et al., Figures S1-2.

	[31]	Storage for up to 5 years at -80⁰C affected lysoPCs, acylcarnitines, hypoxanthine, and serotonin. Details can be found in Yang et al., Figure 3.
Freeze/thaw cycles	[27]	Up to four freeze/thaw cycles affected glycine, methionine, phenylalanine, tryptophan and tyrosine. Details can be found in Anton et al., Supplemental Tables 1 and 2.
	[6]	Up to two freeze/thaw cycles affected methionine sulfoxide, amino acids, PCs, and acetylornithine. Details can be found in Breier et al., Table S7.
	[16]	Up to five freeze/thaw cycles affected choline, glycerol, methanol, and ethanol. Details can be found in Fliniaux et al., Tables 2 and 3B.
	[17]	Up to 10 freeze/thaw cycles affected amino acids. Details can be found in Hirayama et al., Supplemental Figure 6.
	[32]	Up to five freeze/thaw cycles affected lipids, alanine, glucose, acetone, and pyruvate.
	[25]	Up to three freeze/thaw cycles affected arachidonic acid.
	[11]	Up to four freeze/thaw cycles affected carnitine.
		Urine Samples
Centrifugation conditions	[13]	Glutamate increases in samples not centrifuged prior to storage and/or analysis.
	[33]	Centrifugation affected changes in levels observed after four weeks storage at room temperature of acetate, citrate, formate, succinate, trimethylamine, uracil, urea, creatine, creatinine, and phenylacetylglycine. Details can be found in Saude and Sykes, Tables 2 and 3.
Filtration and additives	[33]	Filtration affected changes in levels observed after four weeks storage at room temperature of acetate, benzoate, formate, glycine, Hippurate, lactate, succinate, trimethylamine, uracile, and urea. Details can be found in Saude and Sykes, Tables 2 and 3.
	[33]	Addition of sodium azide affected changes in levels observed after four weeks storage at room temperature of acetate, citrate, formate, glycine, hippurate, lactate, succinate, trimethylamine, uracil, and urea. Details can be found in Saude and Sykes, Table 2.
	[13]	Addition of sodium azide affected urea, succinate, acetate, lactate, and glutamate/glutamine. Details can be found in Bernini et al., Figure S3.
Time delay and temperature	[34]	A delay of one h at room temperature affected succinate.
	[35]	Delays up to 24 h at 9⁰C or 20⁰C affected arginine, valine, leucine/isoleucine, serine, methionine and hexose H1. Details can be found in Rotter et al., Table 1.
	[36]	Delays up to 72 h at room temperature affected creatine, cholic acid, valine, aniline isomer, threonolactone, orotic, trimetnylamine oxide, 2-hydroxy-3-methylbutyric acid, methylguanosine, methylinosine, glutamine, dimethylguanosine, 3-methyl-2-oxovaleric acid, N-acetylcytidine, urobilinogen, urobilin, ketoretinoic acid glucuronide isomer, hydroxyretinoic acid glucuronide isomer, and ascorbic acid. Details can be found in Roux et al., Table 2.
Storage time and temperature	[33]	Storage for four weeks at room temperature affected acetate, benzoate, citrate, creatinine, formate, glycine, hippurate, lactate, malonate, succinate, trimethylamine, and urea. Details can be found in Saude and Sykes, Table 4.
	[37]	Storage for seven days at 4⁰C affected acetate.
Freeze/thaw cycles	[33]	Two freeze/thaw cycles affected urea.
	[35]	Three freeze/thaw cycles affected acylcarnitines and hexose. Details can be found in Rotter et al., Table 4.
Abbreviations: PI, phosphatidylinositol; PC, phosphatidycholine.		

References

1. Denery, J.R., A.A. Nunes, and T.J. Dickerson, *Characterization of differences between blood sample matrices in untargeted metabolomics.* Anal Chem, 2011. **83**(3): p. 1040-7.

2. Nishiumi, S., et al., *Differences in metabolite profiles caused by pre-analytical blood processing procedures.* J Biosci Bioeng, 2018. **125**(5): p. 613-618.
3. Paglia, G., et al., *Influence of collection tubes during quantitative targeted metabolomics studies in human blood samples.* Clin Chim Acta, 2018. **486**: p. 320-328.
4. Wedge, D.C., et al., *Is serum or plasma more appropriate for intersubject comparisons in metabolomic studies? An assessment in patients with small-cell lung cancer.* Anal Chem, 2011. **83**(17): p. 6689-97.
5. Yu, Z., et al., *Differences between human plasma and serum metabolite profiles.* PLoS One, 2011. **6**(7): p. e21230.
6. Breier, M., et al., *Targeted metabolomics identifies reliable and stable metabolites in human serum and plasma samples.* PLoS One, 2014. **9**(2): p. e89728.
7. Townsend, M.K., et al., *Reproducibility of metabolomic profiles among men and women in 2 large cohort studies.* Clin Chem, 2013. **59**(11): p. 1657-67.
8. Carayol, M., et al., *Reliability of Serum Metabolites over a Two-Year Period: A Targeted Metabolomic Approach in Fasting and Non-Fasting Samples from EPIC.* PLoS One, 2015. **10**(8): p. e0135437.
9. Townsend, M.K., et al., *Impact of Pre-analytic Blood Sample Collection Factors on Metabolomics.* Cancer Epidemiol Biomarkers Prev, 2016. **25**(5): p. 823-829.
10. Kamlage, B., et al., *Quality markers addressing preanalytical variations of blood and plasma processing identified by broad and targeted metabolite profiling.* Clin Chem, 2014. **60**(2): p. 399-412.
11. Yin, P., et al., *Preanalytical aspects and sample quality assessment in metabolomics studies of human blood.* Clin Chem, 2013. **59**(5): p. 833-45.
12. Ang, J.E., et al., *Identification of human plasma metabolites exhibiting time-of-day variation using an untargeted liquid chromatography-mass spectrometry metabolomic approach.* Chronobiol Int, 2012. **29**(7): p. 868-81.
13. Bernini, P., et al., *Standard operating procedures for pre-analytical handling of blood and urine for metabolomic studies and biobanks.* J Biomol NMR, 2011. **49**(3-4): p. 231-43.
14. Bervoets, L., et al., *Influence of preanalytical sampling conditions on the ^{1}H NMR metabolic profile of human blood plasma and introduction of the Standard PREanalytical Code used in biobanking.* Metabolomics, 2015. **11**(5): p. 1197-1207.
15. Brunius, C., et al., *Prediction and modeling of pre-analytical sampling errors as a strategy to improve plasma NMR metabolomics data.* Bioinformatics, 2017. **33**(22): p. 3567-3574.
16. Fliniaux, O., et al., *Influence of common preanalytical variations on the metabolic profile of serum samples in biobanks.* J Biomol NMR, 2011. **51**(4): p. 457-65.
17. Hirayama, A., et al., *Effects of processing and storage conditions on charged metabolomic profiles in blood.* Electrophoresis, 2015. **36**(18): p. 2148-2155.
18. Jain, M., et al., *Analytes related to erythrocyte metabolism are reliable biomarkers for preanalytical error due to delayed plasma processing in metabolomics studies.* Clin Chim Acta, 2017. **466**: p. 105-111.
19. Jobard, E., et al., *A Systematic Evaluation of Blood Serum and Plasma Pre-Analytics for Metabolomics Cohort Studies.* Int J Mol Sci, 2016. **17**(12).

20. Kamlage, B., et al., *Impact of Prolonged Blood Incubation and Extended Serum Storage at Room Temperature on the Human Serum Metabolome.* Metabolites, 2018. **8**(1).
21. Malm, L., et al., *Metabolomic Quality Assessment of EDTA Plasma and Serum Samples.* Biopreserv Biobank, 2016. **14**(5): p. 416-423.
22. Midttun, O., et al., *Most blood biomarkers related to vitamin status, one-carbon metabolism, and the kynurenine pathway show adequate preanalytical stability and within-person reproducibility to allow assessment of exposure or nutritional status in healthy women and cardiovascular patients.* J Nutr, 2014. **144**(5): p. 784-90.
23. Trezzi, J.P., et al., *LacaScore: a novel plasma sample quality control tool based on ascorbic acid and lactic acid.* Metabolomics, 2016. **12**(6): p. 96.
24. Wang, Y., et al., *Reproducibility of non-fasting plasma metabolomics measurements across processing delays.* Metabolomics, 2018. **18**(11): p. 129.
25. Wood, J.T., et al., *Comprehensive profiling of the human circulating endocannabinoid metabolome: clinical sampling and sample storage parameters.* Clin Chem Lab Med, 2008. **46**(9): p. 1289-95.
26. Lesche, D., et al., *Does centrifugation matter? Centrifugal force and spinning time alter the plasma metabolome.* Metabolomics, 2016. **12**(10): p. 159.
27. Anton, G., et al., *Pre-analytical sample quality: metabolite ratios as an intrinsic marker for prolonged room temperature exposure of serum samples.* PLoS One, 2015. **10**(3): p. e0121495.
28. Barton, R.H., et al., *High-throughput 1H NMR-based metabolic analysis of human serum and urine for large-scale epidemiological studies: validation study.* Int J Epidemiol, 2008. **37 Suppl 1**: p. i31-40.
29. Moriya, T., Y. Satomi, and H. Kobayashi, *Intensive determination of storage condition effects on human plasam metabolomics.* Metabolomics, 2016. **12**(12): p. 179.
30. Haid, M., et al., *Long-Term Stability of Human Plasma Metabolites during Storage at -80 degrees C.* J Proteome Res, 2018. **17**(1): p. 203-211.
31. Yang, W., et al., *Liquid chromatography-tandem mass spectrometry-based plasma metabonomics delineate the effect of metabolites' stability on reliability of potential biomarkers.* Anal Chem, 2013. **85**(5): p. 2606-10.
32. Pinto, J., et al., *Human plasma stability during handling and storage: impact on NMR metabolomics.* Analyst, 2014. **139**(5): p. 1168-77.
33. Saude, E.J. and B.D. Sykes, *Urine stability for metabolomic studies: effects of preparation and storage.* Metabolomics, 2007. **3**(1): p. 19-27.
34. Budde, K., et al., *Quality assurance in the pre-analytical phase of human urine samples by (1)H NMR spectroscopy.* Arch Biochem Biophys, 2016. **589**: p. 10-7.
35. Rotter, M., et al., *Stability of targeted metabolite profiles of urine samples under different storage conditions.* Metabolomics, 2017. **13**(1): p. 4.
36. Roux, A., et al., *Impact of collection conditions on the metabolite content of human urine samples as analyzed by liquid chromatography coupled to mass spectrometry and nuclear magnetic resonance spectroscopy.* Metabolomics, 2015. **11**(5): p. 1095-1105.
37. Lauridsen, M., et al., *Human urine as test material in 1H NMR-based metabonomics: recommendations for sample preparation and storage.* Anal Chem, 2007. **79**(3): p. 1181-6.
